# Editorial: Insight into plant spatial omics: mass spectrometry imaging

**DOI:** 10.3389/fpls.2023.1273010

**Published:** 2023-08-21

**Authors:** Xiaodong Wang, Jun Han, Zhili Li, Bin Li, Yinglang Wan, Liangyu Liu

**Affiliations:** ^1^ College of Life and Environmental Sciences, Minzu University of China, Beijing, China; ^2^ Key Laboratory of Mass Spectrometry Imaging and Metabolomics (Minzu University of China), State Ethnic Affairs Commission, Beijing, China; ^3^ Genome British Columbia Proteomics Centre, University of Victoria, Victoria, BC, Canada; ^4^ Division of Medical Sciences, University of Victoria, Victoria, BC, Canada; ^5^ Institute of Basic Medical Sciences, Chinese Academy of Medical Sciences and Peking Union Medical College, Beijing, China; ^6^ State Key Laboratory of Natural Medicines, China Pharmaceutical University, Nanjing, China; ^7^ Hainan Key Laboratory for Sustainable Utilization of Tropical Bioresources, College of Tropical Crops, Hainan University, Haikou, China; ^8^ Beijing Key Laboratory of Plant Gene Resources and Biotechnology for Carbon Reduction and Environmental Improvement, and College of Life Sciences, Capital Normal University, Beijing, China

**Keywords:** mass spectrometry imaging, plant science, spatial omics, *in situ* analysis, tissue imaging, endogenous compounds

Plant spatial omics, initiated by mass spectrometry imaging (MSI), is an analytically advanced, label-free technology that is capable of simultaneous determination of the abundances and distribution patterns of endogenous molecules (*e.g.*, proteins, peptides, lipids, primary metabolites and secondary metabolites) directly from the surfaces of thinly-sectioned plant tissues. Accurate characterization of the structure, abundance, and spatial location of these naturally occurring molecules in plants is of paramount importance for comprehensive understanding of plant development, growth, and the interactions between plants and their environment under physiological and pathological conditions or under perturbations caused by stress. Consequently, MSI has gained significant popularity and is increasingly being applied in various areas of plant science such as plant physiology, pathology, resistance to biotic/abiotic stresses, as well as plant-microbe/insect interactions, among others. This Research Topic aims to present a collection of 9 articles that focuses on methodology and application studies related to MSI techniques in plant science, thereby facilitating the dissemination of innovative and groundbreaking insights in the field of MSI-driven plant spatial omics.

The optimization of the process for preparing samples plays a pivotal role in ensuring the achievement of high sensitivity, high spatial resolution, and high-quality signals of MSI. In a groundbreaking study, Qin et al. have described an innovative tissue pretreatment protocol, which is specifically designed for matrix-assisted laser desorption/ionization (MALDI)-MSI analysis of endogenous peptide molecules in castor bean tissues, which are known for their abundance of lipids. Through the utilization of a modified sample washing protocol, the researchers successfully visualized and quantified *Ricinus communis* biomarkers (RCBs) in the tissue sections of castor beans obtained from nine different geographical sources. These findings represent a significant advancement in our understanding of castor bean-related intoxication events, which will shed light on a new research perspective on traceability within this domain.

In a typical MALDI-MSI experiment, it is crucial to select the appropriate MALDI matrix to facilitate the process of analyte ionization. Building upon this premise, Shen et al. have introduced a new approach in which a pre-coated matrix comprised of platinum nanomaterials were prepared in quantities. It was achieved by sputtering platinum nanoparticles onto glass slides using an ion sputterer. By employing the innovative pre-coated matrix of this type, the researchers successfully obtained high-resolution ionic images to depict the spatial distribution of oligosaccharides and lipids in germinating wheat and corn tissue sections through MALDI-MSI. The methodology that Shen et al. proposed presents a fundamental technique that serves as an indispensable tool for exploring the intricate distribution patterns of oligosaccharides and lipids within plant tissues.

The characterization of bioactive secondary metabolites such as alkaloids, sesquiterpenes, and flavonoids using MSI techniques is experiencing substantial growth. This phenomenon can be attributed to the pivotal role of MSI, which plays a crucial role in unraveling the intricacies of plant metabolism, as highlighted by recent findings in the field. In this vein, Liu et al. have made a significant stride by employing a combined approach of ultra-performance liquid chromatography/quadrupole time-of-flight mass spectrometry (UPLC-QTOF-MS) and MALDI-TOF-MSI to analyze the metabolite profiles, spatial distributions, and biosynthetic pathways of various functional metabolites within the stem of *Dendrobium nobile* (*D. nobile*). The investigation successfully identified and relatively quantitated a set of bioactive metabolites, including 11 alkaloids, 10 sesquiterpenes, and 13 other metabolites. It has shed light on the biosynthetic pathways and accumulation patterns of dendrobine in the *D. nobile* stem. Concurrently, Wu et al. have delved into the spatiotemporal distributions of alkaloids in *Gelsemium elegans*, utilizing desorption electrospray ionization MSI. Notably, the study has visualized 23 alkaloids in roots, stems, and leaves of the plant during the seedling stage, while 19 alkaloids were imaged by MS during the mature stage. It has showcased the intriguing phenomena of multiple alkaloid diffusion and transfer within tissues, which correlates with their developmental and maturation processes. Likewise, Liu et al. have successfully undertaken the *in-situ* detection and imaging of the distribution of flavonoids in litchi (*Litchi chinensis*) seed tissue sections via MALDI-MSI, pioneering a groundbreaking work in this field. Notably, the investigation has successfully detected and imaged 15 flavonoid ion signals in the positive ion mode of MS detection. Overall, these studies present essential methodologies for comprehensively understanding the physiological changes occurring in bioactive metabolites and effectively harnessing the potential of plant tissues.

Profiling and imaging strategies using MS techniques have made great contributions to the identification of distinct compounds that exhibit inter- and intra-species differences. In this respect, Liu et al. have made significant advancements by employing MALDI-TOF-MSI to achieve rapid and effective identification at the species level of *Pterocarpus santalinus* and *Pterocarpus tinctorius*. The conducted study successfully screened and identified 15 potential chemical markers, thereby overcoming the limitations caused by traditional methods of identification. The findings of this research hold immense promise in providing vital technical support for conservation of the endangered and valuable wood species. Simultaneously, Liu et al. have also provided insight into the chemical changes of protein-related metabolites derived from *Bombyx batryticatus* (*B. batryticatus*) before and after undergoing the stir-frying process with wheat bran. Their investigations have substantiated that the process of frying not only reduces the toxicity of *B. batryticatus* but also potentially enhances specific biological activities. This highlights the significance of Chinese medicine processing technology. These studies collectively contribute to our understanding of species identification and the chemical transformations that take place during the processing of medicinal substances, thereby paving the way for future advancements in both fields.

The field of plant science has witnessed remarkable progress in recent years, which is primarily driven by advances in rapid and non-destructive optical imaging technology. Notably, these advancements have revolutionized the detection of seed quality, offering unprecedented insights and opportunities. Building upon this trend, Jia et al. have leveraged optical imaging techniques alongside cutting-edge machine learning algorithms to develop a robust model for effectively categorizing the maturity stages and locations of Siberian wild rye (*Elymus sibiricus* L.) seeds. Remarkably, this study has demonstrated that the integration of feature filtering algorithms with machine learning methods yields a highly performant and cost-effective approach for accurately identifying seed maturity statuses. Furthermore, the implementation of the k-means technique to address variations in maturity among plant seeds has further augmented the classification accuracy. These pioneering findings not only contribute to the field of seed quality detection but also showcase the remarkable potential of optical imaging and machine learning in enhancing our understanding of plant development and optimizing agricultural practices.

The investigation of plant-pathogenic microbe interactions utilizing MSI is a burgeoning field in plant sciences. In this context, Maia et al. conducted a detailed MSI analysis of *Vitis vinifera* L. (*V. vinifera*) leaf discs that were infected by *Plasmopara viticola* (*P. viticola*), with the aim to unravel and localize bioactive molecules crucial to the initial stages of pathogen contact with the leaf surface. Notably, the researchers meticulously optimized the preparation of the plant material to overcome the challenges posed by the non-flat nature of *V. vinifera* leaves and the presence of trichomes, which hindered the MS detection. Furthermore, they systematically evaluated different matrices and solvents to acquire high-quality MALDI-MS images. The outcomes of their study unveiled a compelling finding, indicating a conspicuous accumulation of identified sucrose with a non-homogeneous distribution in the infected leaf discs, contrasting with the control samples. Intriguingly, the accumulation of sucrose was predominantly observed around the veins, leading the researchers to develop a hypothesis suggesting the manipulation of sucrose metabolism by the developmental structures of *P. viticola*. This research not only sheds light on the intricacies of plant-microbe interactions but also underscores the significance of MSI in elucidating the dynamic processes underlying host-pathogen relationships in plants. The findings pave the way for further investigations to comprehend the molecular mechanisms at play, which can contribute to the development of innovative strategies for disease control and plant protection.

In summary, this Research Topic presents valuable insights into plant spatial omics through the method development and application of MSI. The articles featured within this Research Topic highlight a wide range of practical or promising applications that are being explored in this field of research and development. Most of the contributors in this Research Topic have concentrated on investigating the spatial distributions of pivotal or distinctive compounds within plant samples through analysis using MSI. It is our earnest desire that these studies will not only improve our understanding of plant spatial omics but also provide groundwork to conduct more extensive investigations on the physiological and biochemical processes of plants.

## Author contributions

XW: Conceptualization, Supervision, Writing – original draft, Writing – review & editing. JH: Conceptualization, Supervision, Writing – review & editing. ZL: Conceptualization, Supervision, Writing – review & editing. BL: Conceptualization, Supervision, Writing – review & editing. YW: Conceptualization, Supervision, Writing – review & editing. LL: Conceptualization, Supervision, Writing – review & editing.

